# Inequality and Social Rank: Income Increases Buy More Life Satisfaction in More Equal Countries

**DOI:** 10.1177/0146167220923853

**Published:** 2020-05-29

**Authors:** Edika G. Quispe-Torreblanca, Gordon D. A. Brown, Christopher J. Boyce, Alex M. Wood, Jan-Emmanuel De Neve

**Affiliations:** 1 University of Oxford, UK; 2 The University of Warwick, Coventry, UK; 3 University of Stirling, UK; 4 London School of Economics and Political Science, UK

**Keywords:** inequality, well-being, income rank, life satisfaction, social class, materialism

## Abstract

How do income and income inequality combine to influence subjective well-being? We examined the relation between income and life satisfaction in different societies, and found large effects of income inequality within a society on the *relationship* between individuals’ incomes and their life satisfaction. The income–satisfaction gradient is steeper in countries with more equal income distributions, such that the positive effect of a 10% increase in income on life satisfaction is more than twice as large in a country with low income inequality as it is in a country with high income inequality. These findings are predicted by an income rank hypothesis according to which life satisfaction is derived from social rank. A fixed increment in income confers a greater increment in social position in a more equal society. Income inequality may influence people’s preferences, such that in unequal countries people’s life satisfaction is determined more strongly by their income.

## Introduction

How does an individual’s income, together with the level of income inequality in the individual’s society, determine how satisfied they are with their lives? Much attention has been given to the economic, psychological, and social consequences of income inequality, which has risen dramatically in many Western (especially English-speaking) countries over recent decades (e.g., Stiglitz, 2012). The adverse health and well-being consequences of rising income inequality are receiving increasing attention in both economics (e.g., Lansley, 2011; Milanovic, 2019; Pontusson, 2005; Stiglitz, 2012) and the social sciences more generally (e.g., Buttrick et al., 2017; Jetten & Peters, 2019; Wilkinson & Pickett, 2009, 2018). Here we explore the interactive effects of income (as an individual-level variable) and income inequality (a society-level variable) on individual life satisfaction.

More specifically, we exploit country-level variation in income inequality to test predictions of the *income rank hypothesis,* according to which an individual’s life satisfaction increases with the relative ranked position of their income within their society. Previous research has shown that people’s self-rated life satisfaction is influenced by the relative ranked position of their income within their social comparison group (Boyce et al., 2010; Brown et al., 2008; Clark et al., 2009). Thus, a person earning an income of US$60 K will be more satisfied with that income if it is the third highest in that person’s social comparison group than they will be if the income of US$60 K is the 10th highest within the comparison group. While recent evidence for effects of income rank on life satisfaction has come from studies *within* individual countries, the income rank hypothesis makes a strong prediction for how the relation between income and life satisfaction should vary *across* countries as a function of the differing income inequality of those countries. Specifically, the income rank hypothesis predicts that the gradient of the relationship between income and life satisfaction will be shallower in countries with more unequal income distributions. This is because a fixed increase in income will move an individual further up the social ladder of incomes in a more equal country, where incomes span a narrower range. To put it another way, in a society with higher income inequality, the income gap that separates any given ranked positions will tend to be larger—and hence the increase in income needed to achieve a given increment in social rank will also be larger. If it is income rank that confers subjective life satisfaction, we would expect that the increase in income needed to achieve a given increment in satisfaction will be smaller in a more equal society than in a more unequal one. In the present article, we test this prediction, using two different large datasets, by examining whether the regression coefficient obtained when predicting life satisfaction from income is larger in more equal countries. We also examine whether the prediction holds for all countries or just for richer countries, as it is possible that the concern for income as a marker of social status, rather than just for the goods and services that it buys, might be more important in richer countries where basic physical needs are already met.

The rest of the paper proceeds as follows. We first note the large literature on the relationship between income and life satisfaction, and then briefly review research that has examined the main effects of income inequality on life satisfaction and other measures of subjective well-being. We then motivate the income rank hypothesis in more detail, and note its prediction that an individual’s income and the inequality of the society they live in should interact in determining life satisfaction. Next, we describe two studies that tested this prediction, each using a different dataset, and show that the slope of the function linking well-being to income is indeed greater in countries where inequality is lower (Study 1 used the World Values Survey integrated questionnaire, and Study 2 used the Gallup World Poll). Finally, we explore the theoretical implications of the results and discuss how they may be reconciled with the widespread assumption that individuals who live in more unequal societies tend to be more materialistic and status-conscious (e.g., Wilkinson & Pickett, 2018).

### Income and Life Satisfaction

A large literature, which we touch on only briefly here, has examined the relationship between income and subjective well-being. Subjective well-being has most often been operationalized as self-reported life satisfaction in econometric studies that have used very large datasets. This literature finds that—within a country at a given time point—individuals with higher incomes have, on average, higher life satisfaction (Easterlin et al., 2010; Stevenson & Wolfers, 2008, 2013). Income’s effect on life satisfaction is, however, greater than its effect on emotional well-being (Kahneman & Deaton, 2010), consistent with the idea that other facets of subjective well-being are not positively associated with, and may even be reduced by, material circumstances (Csikszentmihalyi, 1999; Scitovsky, 1976). Within economics, it is typically further assumed that there is a constant relationship between income and life satisfaction, such that a given increase in income from a fixed starting point produces the same increase in well-being within and across different countries (e.g., Stevenson & Wolfers, 2008). One key aim of the present paper is to show that this assumption of a constant income-satisfaction relationship is incorrect, and that the income-satisfaction relationship varies systematically and predictably across different countries, as predicted by the rank-based account described above.

Other research in both economics and psychology has emphasized the role of social comparison, finding that people gain satisfaction from having a higher income than others (e.g., Clark & Oswald, 1996; Luttmer, 2005). More specifically, according to the income rank hypothesis described earlier, people appear to be sensitive to the *relative ranked position* of their income within a comparison group. Results of several studies support the suggestion that the ranked position of an individual’s or household’s income, rather than the income per se or its relation to a reference income, is beneficial for various types of well-being (Boyce et al., 2010; Brown et al., 2008; Clark & Senik, 2014; Clark et al., 2009; Wood et al., 2012). The income rank hypothesis is also consistent with broader strands of literature, and we return to these below. However, the evidence that rank of income, rather than income, predicts life satisfaction provides the starting point for the present paper.

### Income Inequality and Subjective Well-Being

Intuition—in addition to conventional economic analyses—leads to the expectation of reduced subjective well-being in unequal societies. Especially since Lerner (1944), it has been assumed that redistribution of income from rich to poor, such that inequality is reduced, will increase average well-being because of the diminishing returns of income to well-being at higher levels (see also Yitzhaki, 1979). According to this perspective, the disutility experienced by a wealthy person on losing US$1,000 of income will be less than the utility gain of a poorer person on receiving it.^1^ Indeed, using existing parameters for the income-well-being relationship (Layard et al., 2008), taking 25% of the income of each person in the richest decile of the population of a relatively unequal country (with a Gini coefficient of 45) and sharing it equally amongst all individuals in the poorest decile would increase the well-being of the poorest decile by about 11% while reducing the well-being of the top decile by only about 1%. (Calculation based on numerical simulation assuming a log-normally distributed income distribution with well-being given as $y^{\left(1-p\right)}-1/\left(1-p\right)$ where *y* is income and *p* = 1.26; value taken from Layard et al.)

Despite these economic considerations, empirical studies have often failed to find that income inequality per se is detrimental to mean levels of well-being. Relevant data come from large datasets, with analyses comparing either different countries or different regions within a country. We review these in turn, focusing on effects of inequality on subjective well-being rather than on preferences for redistribution (Alesina & Giuliano, 2010; Ferrer-i-Carbonell & Ramos, 2014) and noting the qualification that people’s subjective perceptions of inequality may be inaccurate (Cruces et al., 2012; Eriksson & Simpson, 2012; Norton & Ariely, 2011; Schneider, 2012).

#### Country-level studies

Recent studies based on larger and combined datasets have converged on the suggestion that income inequality has no discernible effect on subjective well-being in countries with relatively advanced economies, but may be positively associated with well-being in poorer countries (Kelley & Evans, 2017a, 2017b). Earlier studies, often based on small datasets, presented a mixed pattern of results. Thus, some studies have reported no (or negligible) associations between income inequality and various measures of well-being, including life satisfaction (Bjørnskov et al., 2013; Bjørnskov et al., 2008; Diener et al., 1995; Fahey & Smyth, 2004; Zagorski et al., 2014), while others have reported that inequality is beneficial for well-being (Berg & Veenhoven, 2010; Helliwell & Huang, 2008; Ott, 2005), or detrimental for well-being (Alesina et al., 2004; Diener et al., 1995; Fahey & Smyth, 2004; Graham & Felton, 1986; Hagerty, 2000; O’Connell, 2004; Veenhoven, 1984; Verme, 2011).

Many of these studies are cross-sectional rather than longitudinal, and the correlation between inequality and well-being may reverse sign within a given country over time (e.g., in Poland: Grosfeld & Senik, 2010). Mikucka et al. (2017) find that in relatively rich countries there is a positive relationship between subjective well-being and economic growth when the growth is accompanied by reductions in income inequality (see also Oishi & Kesebir, 2015). Moreover, Oishi et al. (2012) found that progressive (and hence inequality-reducing) taxation is associated with increased national well-being (see also Oishi et al., 2018).

In summary, cross-national studies have failed to find a consistent and substantial detrimental effect of income inequality on subjective well-being, although findings are mixed.

#### Within-country studies

Within-country studies have also produced mixed results. Some studies have found negligible or no effects of regional income inequality on well-being (Alesina et al., 2004; Senik, 2004), while others have found either positive (Clark, 2003; Jiang et al., 2012) or negative (Blanchflower & Oswald, 2003; Hagerty, 2000; Morawetz et al., 1977; Oshio & Kobayashi, 2010; Schwarze & Härpfer, 2007; Tomes, 1986) effects.

Within-country effects might be more difficult to interpret than across-country effects, as the presence of high incomes may increase well-being if it acts as a signal to lower earners that their own situation may improve—a “tunnel effect” (Hirschman & Rothschild, 1973). Senik (2004), using Russian data, found no effect of regional inequality but obtained a positive effect of reference group income on well-being and concluded that the data were consistent with an effect of this type (see also Clark et al., 2009; Eggers et al., 2006; Hirschman & Rothschild, 1973). Mediating variables may also be important: Oishi et al. (2011) examined the relation between inequality and happiness over nearly four decades within the United States, and found that greater inequality led to reduced happiness with the relationship being mediated by levels of trust for most income groups (see also Cheung & Lucas, 2016; Delhey & Dragolov, 2014; Oishi et al., 2018). Attitudes toward fairness and inequality may also matter (Alesina et al., 2004; Buttrick & Oishi, 2017; Napier & Jost, 2008; Schneider, 2012).

In the light of these issues, and the fact that our own study focuses on the role of cross-country rather than within-country differences in inequality, we do not consider these within-country studies further and turn instead to our main hypothesis.

### Rank-Based Social Comparison, Income, and Inequality

We have reviewed the literature showing that (a) an individual’s life satisfaction is better predicted by the relative ranked position of their income than by their income and (b) there is little consistent evidence for any substantial detrimental effect of income inequality on country-level well-being. These results accord well with the income rank hypothesis. We note in particular that the mean relative ranked position of individuals within a society will always be .5, and that if life satisfaction is determined solely by ranked position there can by definition be no direct effect of income inequality on mean life satisfaction.

The income rank hypothesis also fits well with the wider literature. A rank-based approach resonates with the idea that the desire for status is important for people (Anderson et al., 2015). A concern for rank could be intrinsic (Frank, 2010) or could reflect the rank-based allocation of rewards in many aspects of life (Cole et al., 1992). Concerns with social rank appear closely related to both brain activity and well-being: Social comparison affects reward related brain activity (Fliessbach et al., 2007), social rank affects stress in both humans and animals (Sapolsky, 2005), and stress-related cortisol levels are associated specifically with social evaluative threats (Dickerson & Kemeny, 2004). Moreover, a concern with relative rank is consistent with cognitive models which suggest that subjective judgments of economic quantities (such as income) are influenced by the relative ranked position of the quantity within a context (Bhui & Gershman, 2018; Parducci, 1995; Stewart et al., 2006).

The aim of the present paper is, therefore, to test the novel prediction of the income rank hypothesis, as outlined in the Introduction, that the gradient of the relationship between income and life satisfaction will be steeper in countries with more equal income distributions.

## Study 1

### Method

We start by focusing on the associations between log(income) and life satisfaction within countries and on the critical issue of whether those associations vary with country-level income inequality. In the first study, we based our estimates on the most recent longitudinal data available from the World Values Survey integrated questionnaire (WVS: http://www.worldvaluessurvey.org; dataset: WVS_Longitudinal_1981-2014_rdata_v_2015_04_18). WVS measures life satisfaction through a 1 to 10 scale question “All things considered, how satisfied are you with your life as a whole these days?,” where 1 means you are “completely dissatisfied” and 10 means you are “completely satisfied.”

Gini coefficients were used as the measure of income inequality, and were taken from the Standardized World Income Inequality Database (SWIID: Solt, 2016). We used net Gini measures from the year preceding the life satisfaction survey for each country (or, if absent, from the prior year). We included in our analyses only countries for which Gini coefficients were available from the SWIID.

**Figure 1.**
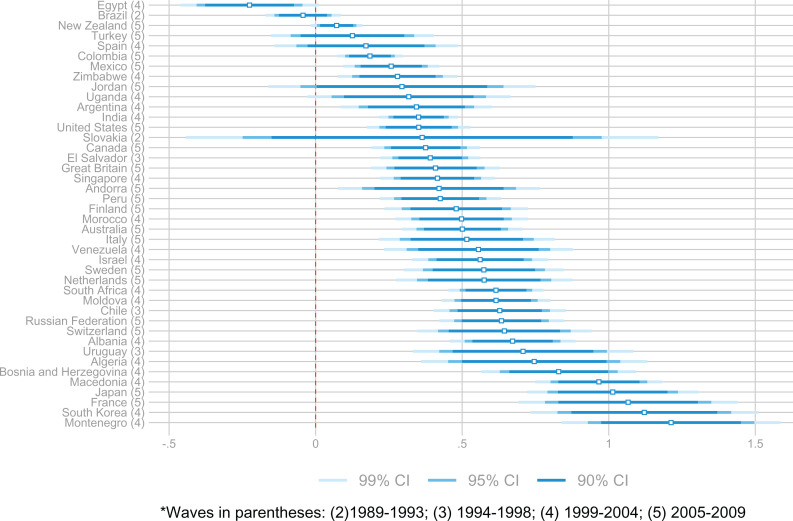
Within-country life satisfaction-income gradient using WVS data. *Note.* The data include 42 countries and the most recent survey with life satisfaction and income data available. Life satisfaction ranges from 1 to 10. OLS estimates control for gender, employment, a four-degree polynomial of age, and the interaction of this polynomial with gender. WVS = World Values Survey; OLS = ordinary least squares.

For each country, we used the most recent year with usable data available in the longitudinal WVS integrated questionnaire. We used only a single year for each country to avoid collinearity issues associated with the use of country and year dummies (Verme, 2011). Although the WVS includes socioeconomic data for 101 countries, income levels are reported for only 44 countries. After excluding countries for which Gini coefficients were unavailable, we were left with a remaining sample of 42 countries (displayed in Figure 1).

Observations in this set of 42 countries can be represented as a hierarchical, multilevel structure, where level 1 units are the individuals and level 2 units are the countries. Our main focus is on whether the effect of individual-level income on subjective life satisfaction can be explained by country-level inequality differences. Equations 1 and 2 describe the general two-level representation of this multilevel structure:

1 $$Life\;Satisfaction_{ict}={\text{α}}_{ct}+{\text{β}}_{ct}Ln(Income_{ict})+\text{ψ}X_{ict}+\;{\text{∊}}_{ict}$$

2 $${\hat{\text{β}}}_{ct}=\text{γ}+\text{η}Gini_{c\;t-1}+{\text{λ}}_{1}GDP_{ct}+{\text{λ}}_{2}GDP_{ct}^{2}+{\text{λ}}_{3}GDP_{ct}^{3}+{\text{ν}}_{ct}$$

In Equation 1, the level of observations is the individual *i* in country *c* and year *t*. The independent variable of interest is the natural log of household income $Ln(Income_{ict})$. Matrix $X_{ict}$ includes a vector of individual demographic controls. Because income is measured in log terms, the coefficient $(\hat{{\text{β}}_{ct}})/100$ represents the increase in life satisfaction following a 1% rise in income. Note that the coefficient ${\text{β}}_{ct}$ in Equation 1 allows for variation in the income-life satisfaction relationship across countries. In Equation 2, this variation is modeled as a function of two country-level indicators, the Gini index and the gross domestic product (GDP) per capita (at purchasing power parity). We also included controls for the linear, square, and cubic terms of GDP per capita to account fully for the possibility that a percentage increase in income will have different effects on life satisfaction in wealthier countries compared with poorer ones.

Both equations could be estimated simultaneously under the assumption that the individual-level effects in ψ do not vary across countries and years and that the variation in the parameters across level 2 units (Gini index and GDP per capita) can be characterized by a normal distribution. However, rather than pooling the data and estimating Equations 1 and 2 simultaneously, we follow a two-step estimation procedure. As a first step, we estimate the marginal effect of income on life satisfaction, using the linear model described in Equation 1, for each level 2 unit. As a second step, we use these estimated parameters as dependent variables for the country-level regression described in Equation 2. The two-step procedure is a multilevel method that provides a very flexible specification. It allows for different individual-level effects across countries and years in ψ, and does not impose any further distributional assumption on the level-2 parameters. The two-step procedure therefore accommodates the (reasonably large) cross-country cultural differences in life satisfaction and its determinants that we would expect in the WVS data.

While the estimation procedure is straightforward, the estimations of Equations 1 and 2 require some comment. In Equation 1, the independent variable of interest is the natural log of household income, but the WVS reports income in categories with lower and upper bounds. To obtain a continuous variable, for each country we fitted interval regressions to the income data under the assumption that income is log-normally distributed (following the approach adopted by Stevenson and Wolfers (2013), who estimated the effect of income on life satisfaction using WVS surveys conducted in 48 countries in the period 1999-2004).^2^ In addition, matrix $X_{ict}$ includes the same demographic controls that Stevenson and Wolfers used: gender, a quartic polynomial for age, and the interactions between gender and the age polynomial. We additionally included controls for the employment status of i  with a set of dummies distinguishing full-time worker, part-time worker, self-employed, retired, housewife, student, unemployed, and other. We included only adult respondents in our sample (individuals >18 years old).

To account for the uncertainty in the estimates of ${\text{β}}_{ct}$ and enable valid inferences, we estimated Equation 2 via feasible generalized least square estimators (FGLS) as set out by Lewis and Linzer (2005). Thus, we weighted each observation in Equation 2 by the inverse of (${\text{σ}}^{2}+{\text{ω}}_{c}^{2}$), where ${\text{σ}}^{2}$ is the variance of the component of the regression residual that is not due to sampling of the dependent variable and ${\text{ω}}_{c}^{2}$ is the variance of sampling error in the dependent variable ${\hat{\text{β}}}_{ct}$ (estimated via Equation 1).

### Results

Descriptive statistics of the sample of the study are displayed in the Supplemental Material (Table A1). The average age of the individuals in the sample is 41 years. Approximately 49% of the individuals are male, 37% are employed full-time, 19% are either self-employed or employed part-time, and 9% are unemployed. Table A1 also displays some initial evidence of a relationship between income and life satisfaction. We observe that the average measures of life satisfaction are higher in countries belonging to the third tercile of GDP per capita.

Our estimates of the marginal effect of individual log(income) on individual life satisfaction across countries are displayed in Figure 1 (${\hat{\text{β}}}_{ct}$ as described by Equation 1). These parameter estimates imply that, in most countries, income has a strong positive effect on individuals’ satisfaction with their lives. This result, while not the primary focus of the present paper, is consistent with the previous literature.

**Figure 2.**
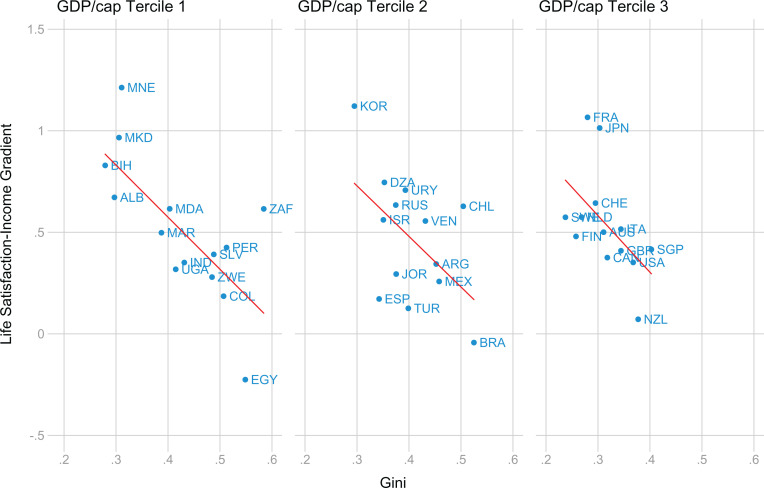
Relation between income inequality and the within-country life satisfaction-income gradient using WVS data. *Note.* The data include 42 countries and the most recent survey with life satisfaction and income data available. Panels are divided into three terciles based on GDP/cap values (in US$10,000 − PPP, 2011). WVS = World Values Survey; GDP = gross domestic product.

Turning to the main hypothesis of interest, Figure 2 plots the relationship of our estimates to the countries’ income inequality levels, separately for terciles based on GDP per capita, as it is possible that the concern for income as a marker of social status, rather than just for the goods and services that income buys, might be more important in richer countries where basic physical needs are already met. The inclusion of GDP also reflects the fact that, because income is measured in log terms, the coefficient $(\hat{{\text{β}}_{ct}})/100$ represents the increase in life satisfaction following a 1% rise in income. A percentage increase in income might have a different effect on life satisfaction in wealthier countries compared with poorer ones, because a 1% rise in income is in absolute terms larger in wealthier countries.

The figure shows a strong relationship, *r*(42) = −.47, *p =* .0017, for the underlying data), such that a 10% increase in income has a positive effect on life satisfaction that is substantially larger in low-inequality countries. There appears to be little effect of per capita GDP on this relationship.

Table 1 reports formal tests of the relationship observed in Figure 2. Estimates correspond to the model described by Equation 2. We observe in Column 1 a significant coefficient for the effect of Gini. The coefficient is negative, showing that income-satisfaction coefficients are larger when income inequality is lower as predicted by the income rank hypothesis. Since a rise in income in one percentage point in low-inequality countries (which are typically richer) is not equal to a rise of the same magnitude in high-inequality countries, we included in Column 2 the linear, square, and cubic terms of GDP per capita (at purchasing power parity). The marginal effect of the Gini index remained negative and significant at 1%.

**Table 1.** Relation Between Income Inequality (Gini) and the Within-Country Life Satisfaction-Income Gradient (WVS data).

Variables	All countries	
	(1)	(2)
	FGLS	FGLS
Gini index (0–1 scale)	−1.556**	−2.704***
	[−2.632, −0.479]	[−4.114, −1.295]
GDP/cap (in US$10,000 − 2011 PPP)		
GDP/cap		0.320
		[−0.0648, 0.704]
GDP/cap^2^		−0.172*
		[−0.335, −0.00804]
GDP/cap^3^		0.0205*
		[0.00126, 0.0398]
Constant	1.084***	1.493***
	[0.668, 1.501]	[0.872, 2.114]
Observations	42	40
*R* ^2^	.236	.414
σ	.245	.224
ω	.102	.0964

*Note*. Columns show FGLS. Data include the most recent wave with available satisfaction and income data in the WVS. The dependent variable is the (within country) life satisfaction-income gradient ($\hat{\text{β}}$) shown in Figure 1. The unit of observation is a country. σ denotes the standard deviation of the component of the regression residual that is not due to sampling of the dependent variable, while ω represents the standard deviation of sampling error in the dependent variable. 95% confidence intervals in brackets. WVS = World Values Survey; FGLS = feasible generalized least square estimators; GDP = gross domestic product.

*p < .05. **p < .01. ***p < .001.

Although Figure 2 shows little evidence that the relationship of interest (i.e., between inequality and the income-satisfaction gradient) is different in wealthier nations, we nevertheless tested for this interaction. We re-estimated the models including the interaction between Gini and GDP per capita in the second step of our two-step estimation procedure. This analysis, as expected, revealed a null effect for this interaction (*B* = 0.132, 95% [−0.537, 0.801]).

The above analyses focus directly on the predictions of the income rank hypothesis. In response to the suggestion of a referee,^3^ we also tested the hypothesis that there might be a greater divergence between measures of social class and income in relatively equal (vs. unequal) countries. Subjective social class is available in the WVS for 33 countries of our sample (the Gallup World Poll dataset, used in Study 2 below, does not incorporate a measure of social class). We replicated our main analysis but replaced our measure of life satisfaction by the individuals’ subjective reports of their social class. We then tested whether the effect of income on subjective social class is larger in countries with more equal income distributions, that is, whether the increase in income needed to achieve a given increment in the social class hierarchy will be smaller in more equal countries.

**Figure 3.**
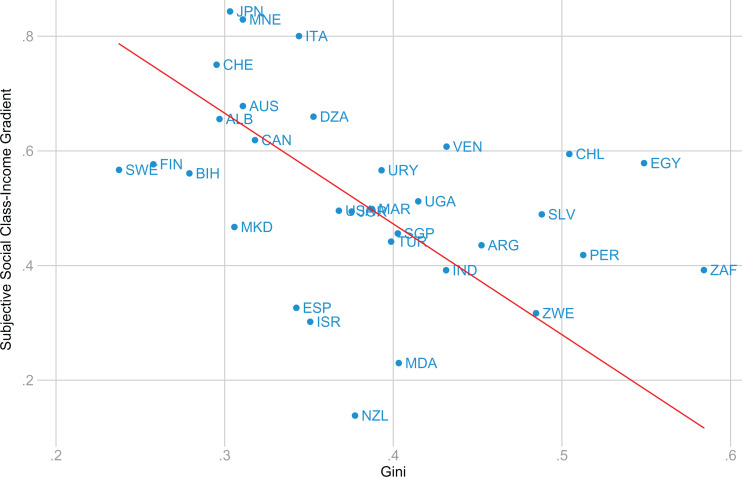
Relation between income inequality and the within-country subjective social class-income gradient using WVS data. *Note*. The data include the subset of countries from the main analysis with available subjective social class data in the WVS (33 countries). WVS = World Values Survey.

To make the analysis comparable to that performed with life satisfaction, we recoded the variable to an *increasing* five-point scale where 1 means “lower class” and 5, “upper class” (survey questions are described in Table A4 [Supplemental Material]). Figure 3 suggests that the income-social-class gradient is indeed larger in countries with more equal income distributions, and Table A2 (Supplemental Material) shows that the effect of the Gini coefficient on the gradient remains significant (this analysis included the same set of controls for GDP per capita as were used in our main analysis).

### Discussion

Study 1 tested the key prediction of the income rank hypothesis and found, as predicted, that a fixed increase in income buys a greater increase in life satisfaction in more equal countries. In the main analysis, for example, the effect of a 10% increase in income on life satisfaction is 2.5 times larger for a low (5th percentile) inequality country than it is for a high (95th percentile) country. The key result did not vary significantly with country wealth, and was also found when self-reported social class was used (instead of life satisfaction) as the key dependent variable.

Although we used the most recent WVS longitudinal data available to produce the most recent country level estimates, because of the absence of usable individual income data for a number of countries our life satisfaction-income gradient estimates are based on different survey years. Moreover, limited control variables are available. Other datasets (such as the Gallup World Poll dataset that we analyze below) contain measures of corruption and confidence in institutions which allow this possible omitted country-level variable bias to be addressed. For robustness, and to address the concern that our estimates might reflect particular country differences related to the time at which surveys were administered, we conducted Study 2.

## Study 2

In Study 2 we explored whether the predicted effect of inequality on the income-well-being relation holds within a much larger and more diverse set of countries than in Study 1. We used data from the Gallup World Poll. The Gallup World Poll is a large-scale repeated cross-sectional household survey covering more than 150 countries across different waves. We studied 76 countries with available well-being and income data for the period 2009-2018. We analyzed four waves spaced by 2 years: Wave 12, 2017-2018, Wave 10, 2015-2016, Wave 7, 2012-2013, and Wave 4, 2009-2010. Overall, 362,274 data points were available for the analysis reported below.

The Gallup World Poll evaluates subjective well-being using the standard Cantril Self-Anchoring Striving Scale (Cantril, 1965). Participants respond to the question: “Please imagine a ladder, with steps numbered from 0 at the bottom to 10 at the top. The top of the ladder represents the best possible life for you and the bottom of the ladder represents the worst possible life for you. On which step of the ladder would you say you personally feel you stand at this time?.” In addition, other different questions are designed to capture various other dimensions of emotional well-being, allowing us to evaluate whether inequality changes the relation between income and measures of positive effect (optimism and enjoyment) as well as measures of negative affect (anger, worry, and stress).

### Method

The analysis adopted the same two-step procedure as was used in Study 1. However, in Study 2, which uses the Gallup World Poll data, we were able to add an initial approximation of the overall main effect of inequality on life satisfaction before our formal estimation procedure. This approximation pools all observations across countries and years and assumes that the effect of all individual-level controls is fixed across these two dimensions—thus, this approximation ignores country-level heterogeneity.^4^

As in the earlier study, we included controls for age, gender (a four-degree polynomial of age and its interaction with gender) and employment status. We additionally included demographic controls for education, marital status, self-reported health, urban/rural areas, and fixed effects for the survey years. Also, as in the earlier study, we used net Gini values for the year preceding the survey waves. This exercise allowed us to introduce an overall estimate of the main effect of inequality on life satisfaction. However, because these initial results will mask the country-level differences that are of primary interest to our hypothesis, we next computed FGLS estimators following the two-step procedure described by Equations 1 and 2, thus estimating different coefficients for each country and wave and retaining the full set of richer controls. As a robustness test, we also computed the income coefficient of variation for each country and wave as an alternative measure of inequality and repeated our main analysis.

Finally, to evaluate whether income inequality moderated the relation between income and other measures of emotional well-being, we repeated our estimation strategy but replacing life satisfaction by measures of positive effect (optimism and enjoyment) as well as measures of negative affect (anger, worry, and stress). Table A5 (Supplemental Material) details the survey questions used to measure these other facets of well-being.

### Results

Descriptive statistics for the Study 2 sample are displayed in Table A3 (Supplemental Material). The average age of the individuals in the sample is 44 years. Approximately 44% of the individuals are male, 27% are employed full-time, and 53% are married. Only 32% of them come from a large city, and most of them (54%) completed secondary education. As in Study 1, we observe a positive relationship between income and life satisfaction, with countries in the fourth quartile of GDP per capita displaying higher average measures of life satisfaction.

Table 2 displays the linear regression estimates of the main effects of income and inequality on life satisfaction by pooling all individual observations across countries and waves. Turning to the key prediction of the income rank hypothesis, despite the richer set of controls, Column 3 shows the predicted negative and significant interaction between Gini and log(income), such that the effect of income on life satisfaction was smaller for individuals living in countries with higher income inequality. The results also suggest an association between life satisfaction and income inequality (i.e., a positive main effect of income inequality on satisfaction) as well as the expected main effect of income on life satisfaction. However, because these associations could mask country-level heterogeneity, we focus on the interaction of interest and estimated FGLS estimators following the two-step procedure described in Equations 1 and 2.

**Table 2.** Relation Between Income and Life Satisfaction.

Variables	(1)	(2)	(3)
	OLS	OLS	OLS
Ln income	0.603***	0.643***	1.239***
	[0.532, 0.674]	[0.568, 0.719]	[0.894, 1.584]
Gini index (0–1 scale)		1.476	16.40***
		[−0.384, 3.337]	[8.204, 24.59]
Ln income # Gini index			−1.602***
			[−2.395, −0.809]
Gender = female	−1.087**	−1.085**	−1.062**
	[−1.734, −0.440]	[−1.730, −0.439]	[−1.713, −0.411]
*Employment status (Ref: Employed full-time for an employer* **)**			
Employed full-time for self	−0.0503	−0.0724	−0.0569
	[−0.136, 0.0358]	[−0.162, 0.0174]	[−0.145, 0.0309]
Employed part-time do not want full-time	0.208***	0.201***	0.191***
	[0.129, 0.288]	[0.121, 0.282]	[0.114, 0.269]
Unemployed	−0.585***	−0.598***	−0.590***
	[−0.690, −0.479]	[−0.702, −0.495]	[−0.689, −0.490]
Employed part-time (want full-time)	−0.0952*	−0.115**	−0.124**
	[−0.176, −0.0148]	[−0.198, −0.0330]	[−0.203, −0.0436]
Out of workforce	−0.0969*	−0.101**	−0.0802*
	[−0.171, −0.0231]	[−0.174, −0.0289]	[−0.150, −0.0106]
Refused to answer/missing	−0.292	−0.284	−0.249
	[−0.622, 0.0380]	[−0.606, 0.0371]	[−0.574, 0.0755]
*Marital status (Ref: Single/never been married)*			
Married	−0.0757	−0.0548	−0.0471
	[−0.162, 0.0109]	[−0.133, 0.0238]	[−0.125, 0.0310]
Separated	−0.0228	−0.0341	−0.0403
	[−0.133, 0.0875]	[−0.137, 0.0689]	[−0.143, 0.0621]
Divorced	−0.214***	−0.166***	−0.145**
	[−0.318, −0.109]	[−0.258, −0.0746]	[−0.235, −0.0554]
Widowed	−0.296***	−0.272***	−0.249***
	[−0.401, −0.190]	[−0.372, −0.173]	[−0.349, −0.150]
Domestic partner	0.237**	0.213*	0.170*
	[0.0767, 0.397]	[0.0473, 0.379]	[0.00771, 0.333]
Refused to answer/missing	0.313*	0.336**	0.345**
	[0.0699, 0.556]	[0.103, 0.569]	[0.135, 0.556]
*Rural/urban area (Ref: Rural area or on a farm)*			
A small town or village	0.136*	0.133*	0.122*
	[0.0256, 0.246]	[0.0272, 0.240]	[0.0145, 0.229]
A large city	0.172*	0.147*	0.172*
	[0.0213, 0.323]	[0.00541, 0.288]	[0.0381, 0.307]
A suburb of a large city	0.196*	0.171*	0.156*
	[0.0356, 0.357]	[0.0119, 0.330]	[0.00268, 0.310]
Refused to answer/missing	0.542*	0.496*	0.474*
	[0.105, 0.979]	[0.0783, 0.915]	[0.0401, 0.907]
*Education (Ref: Completed elementary education or less)*			
Secondary	0.397***	0.413***	0.416***
	[0.287, 0.507]	[0.308, 0.518]	[0.310, 0.522]
Completed 4 years of education beyond high school.	0.710***	0.723***	0.721***
	[0.570, 0.849]	[0.587, 0.858]	[0.588, 0.854]
Refused to answer/missing	0.655***	0.683***	0.676***
	[0.448, 0.862]	[0.487, 0.878]	[0.480, 0.871]
*Physical health near-perfect (Ref: Rate 1 Strongly disagree)*			
Rate 2	0.409***	0.412***	0.431***
	[0.249, 0.569]	[0.253, 0.570]	[0.285, 0.578]
Rate 3	0.753***	0.751***	0.763***
	[0.601, 0.905]	[0.602, 0.901]	[0.628, 0.899]
Rate 4	1.092***	1.083***	1.094***
	[0.919, 1.264]	[0.912, 1.253]	[0.933, 1.254]
Rate 5: Strongly agree	1.285***	1.266***	1.287***
	[1.098, 1.473]	[1.084, 1.447]	[1.119, 1.455]
Refused to answer/missing	1.127***	1.146***	1.169***
	[0.766, 1.488]	[0.785, 1.506]	[0.808, 1.529]
Constant	2.296***	1.367*	–4.423*
	[1.354, 3.238]	[0.00214, 2.731]	[–8.118, –0.728]
Year FEs	Yes	Yes	Yes
Age (four-degree polynomial) and its interaction with gender	Yes	Yes	Yes
Observations	362,274	362,274	362,274
*R* ^2^	.184	.186	.189

*Note*. The table provides an initial analysis of the effect of income and income inequality on life satisfaction. Life satisfaction scores range from 0 to 10. Data include 76 countries across four waves: Wave 12, 2017-2018, Wave 10, 2015-2016, Wave 7, 2012-2013, and Wave 4, 2009-2010. The unit of observation is an individual × country × year. Columns show OLS estimators with standard errors clustered by country. The dependent variable is the (within country and year) individual life satisfaction score described by Equation 1. All models include FEs for the survey years, a four-degree polynomial of age, and the interaction of this polynomial with gender. 95% confidence intervals in brackets. OLS = ordinary least squares.

*p < .05. **p < .01. ***p < .001.

**Figure 4.**
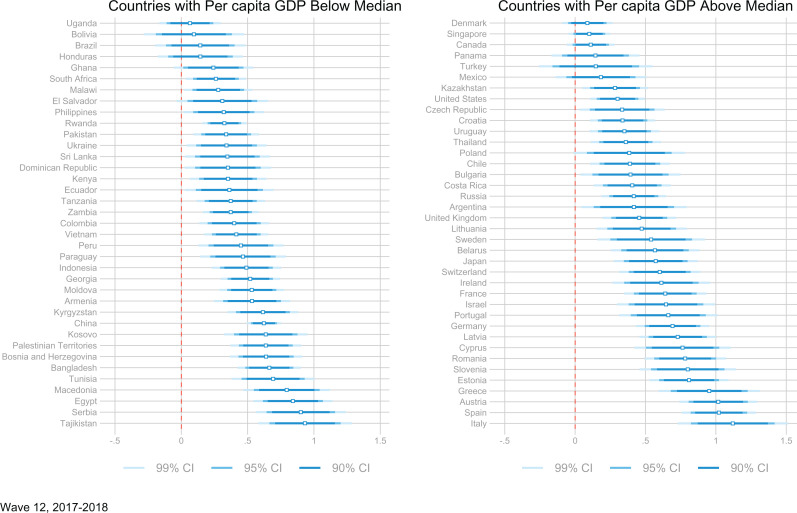
Within-country life satisfaction-income gradient for wave 12 (2017-2018). *Note.* GDP = gross domestic product.

The coefficients relating log(income) to life satisfaction for the wave 2017-2018 are plotted in Figure 4. We observe considerable heterogeneity in the size of the coefficients across countries. However, in most countries the effect of log(income) on life satisfaction is positive and significant. Figures A1 and A2 in the Supplemental Material show the remaining coefficients for the other three waves. Across the four waves, the effect size of log(income) appears to be stable within countries.

**Figure 5.**
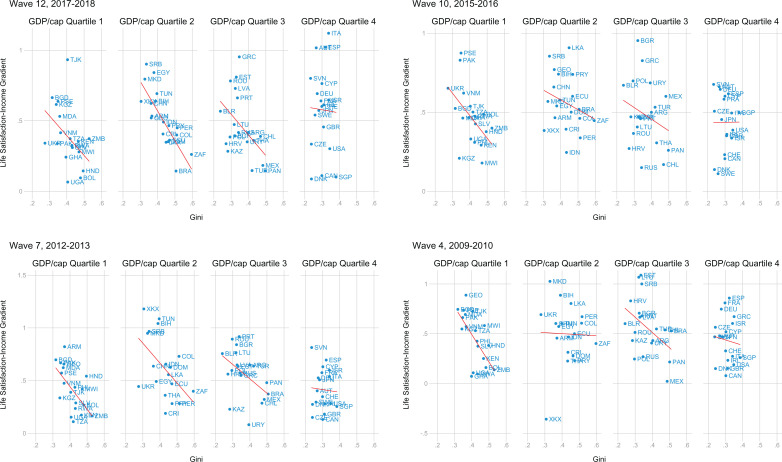
Relation between income inequality and the within-country life satisfaction-income gradient (described in Figures 1 and 2). Panels are divided into four quartiles based on GDP/cap values for each survey year (in US$10,000 − PPP, 2011). *Note.* GDP = gross domestic product.

Figure 5 displays the relation between these coefficients and the Gini index. Countries are divided by quartiles of GDP per capita. The figure suggests that the association with the Gini index may be stronger in low-income countries.

Table 3 presents the results of the two-step estimation procedure and reveals the predicted effect of Gini on the life satisfaction–income gradient, such that income’s effects on life satisfaction are greater in more equal countries. This effect appears higher in magnitude for low-income countries, consistent with Figure 5, and does not reach significance for the richest quartile of countries. It is noteworthy that the range of Gini values is rather narrow for the richest quartile of countries, reflecting in part our use of net rather than gross Gini measures and making any relationship more difficult to observe. The three-way interaction between individual income, country Gini, and GDP per capita was, however, nonsignificant (*B* = 0.337, 95% CI = [−0.0488, 0.723]).

**Table 3.** Relation Between Income Inequality (Gini) and the Within-Country Life Satisfaction-Income Gradient.

Variables	All Countries		GDP/cap Quartile 1		GDP/cap Quartile 2		GDP/cap Quartile 3		GDP/cap Quartile 4	
	(1)	(2)	(3)	(4)	(5)	(6)	(7)	(8)	(9)	(10)
	FGLS	FGLS	FGLS	FGLS	FGLS	FGLS	FGLS	FGLS	FGLS	FGLS
Gini index (0-1 scale)	−0.630*	−0.980***	−1.561**	−1.432**	−1.038*	−1.236*	−1.389*	−1.378*	−0.154	0.439
	[−1.129, −0.131]	[−1.489, −0.471]	[−2.461, −0.661]	[−2.306, −0.558]	[−2.042, −0.0349]	[−2.408, −0.0653]	[−2.522, −0.256]	[−2.414, −0.342]	[−2.607, 2.298]	[−1.497, 2.375]
GDP/cap (in US$10,000 − 2011 PPP)										
GDP/cap		0.168*		2.416		12.47		−2.015		−1.331
		[0.0366, 0.299]		[−2.377, 7.208]		[−2.988, 27.92]		[−10.61, 6.579]		[−3.201, 0.538]
GDP/cap^2^		−0.0659**		−4.501		−10.36		0.699		0.224
		[−0.110, –0.0223]		[−16.12, 7.120]		[−24.25, 3.521]		[−3.328, 4.727]		[−0.117, 0.566]
GDP/cap^3^		0.00532**		2.560		2.799		–0.0670		–0.0125
		[0.00161, 0.00904]		[−5.908, 11.03]		[−1.274, 6.873]		[−0.686, 0.552]		[−0.0319, 0.00694]
Constant	0.580***	0.786***	1.013***	0.592	0.960***	−3.804	0.991***	2.690	0.561	2.765
	[0.339, 0.822]	[0.523, 1.050]	[0.654, 1.373]	[−0.108, 1.291]	[0.506, 1.414]	[−9.508, 1.899]	[0.503, 1.479]	[−3.327, 8.707]	[−0.0852, 1.208]	[−0.371, 5.901]
Year *FE*	Yes	Yes	Yes	Yes	Yes	Yes	Yes	Yes	Yes	Yes
Observations	298	298	76	76	74	74	76	76	72	72
*R* ^2^	.0455	.133	.228	.284	.127	.175	.174	.211	.0841	.309
σ	.211	.200	.170	.167	.217	.215	.191	.192	.215	.185
ω	.106	.106	.0923	.0923	.107	.107	.119	.119	.106	.106

*Note*. Columns show FGLS with standard errors clustered by country. The dependent variable is the (within country and year) life satisfaction-income gradient ($\hat{\text{β}}$) described by Equation 2. The unit of observation is a country × year. σ denotes the standard deviation of the component of the regression residual that is not due to sampling of the dependent variable, while ω represents the standard deviation of sampling error in the dependent variable. 95% confidence intervals in brackets. GDP = gross domestic product; FGLS = feasible generalized least square estimators.

*p < .05. **p < .01. ***p < .001.

In Table 4, we present for robustness an analysis using the income coefficient of variation as an alternative measure of income inequality. Figure A3 in the Supplemental Material compares its distribution with that of the Gini coefficient and shows a higher degree of skewness for the coefficient of variation (even after dropping extreme outliers above the 95 percentile of the coefficient of variation). Despite their different distributions, Table 4 shows qualitatively similar results to those found using the Gini coefficient, with a clear overall effect, although in this case the effect was significant for quartiles one and four but not two or three. As when inequality was measured with Gini coefficients, we found that the three-way interaction between individual income, country income coefficient of variation, and GDP per capita was nonsignificant (*B* = −0.000558, 95% CI = [−0.0270, 0.0259]).

**Table 4.** Relation Between Income Inequality (Coefficient of Variation for Income) and the Within-Country Life Satisfaction-Income Gradient.

Variables	All countries		GDP/cap Quartile 1		GDP/cap Quartile 2		GDP/cap Quartile 3		GDP/cap Quartile 4	
	(1)	(2)	(3)	(4)	(5)	(6)	(7)	(8)	(9)	(10)
	FGLS	FGLS	FGLS	FGLS	FGLS	FGLS	FGLS	FGLS	FGLS	FGLS
Coefficient of variation	−0.153***	−0.138***	−0.162**	−0.140*	−0.148	−0.133	−0.0848	−0.0809	−0.217***	−0.182**
	[−0.218, −0.0882]	[−0.204, −0.0726]	[−0.269, −0.0553]	[−0.249, −0.0304]	[−0.345, 0.0479]	[−0.335, 0.0681]	[−0.260, 0.0902]	[−0.270, 0.108]	[−0.324, –0.111]	[−0.300, –0.0631]
GDP/cap (in US$10,000 − 2011 PPP)										
GDP/cap		0.0778		2.483		10.47		−2.098		−0.393
		[−0.0513, 0.207]		[−2.467, 7.433]		[−2.725, 23.67]		[−11.75, 7.558]		[−1.991, 1.205]
GDP/cap^2^		−0.0244		−5.109		−9.112		0.794		0.0599
		[−0.0643, 0.0155]		[−17.23, 7.010]		[−21.11, 2.889]		[−3.722, 5.309]		[−0.226, 0.346]
GDP/cap^3^		0.00157		3.253		2.555		−0.0860		−0.00341
		[−0.00163, 0.00477]		[−5.537, 12.04]		[−1.020, 6.129]		[−0.778, 0.606]		[−0.0194, 0.0126]
Constant	0.535***	0.516***	0.580***	0.196	0.585***	−3.285	0.613***	2.250	0.667***	1.641
	[0.440, 0.629]	[0.385, 0.647]	[0.390, 0.770]	[−0.471, 0.862]	[0.426, 0.744]	[−8.015, 1.445]	[0.292, 0.934]	[−4.503, 9.003]	[0.600, 0.734]	[−1.006, 4.288]
Year FE	Yes	Yes	Yes	Yes	Yes	Yes	Yes	Yes	Yes	Yes
Observations	284	284	71	71	71	71	76	76	66	66
*R* ^2^	.101	.121	.168	.199	.108	.137	.0537	.0981	.309	.425
σ	.201	.199	.179	.180	.220	.222	.211	.211	.170	.155
ω	.108	.108	.0936	.0936	.108	.108	.119	.119	.111	.111

*Note*. Columns show FGLS with standard errors clustered by country. The dependent variable is the (within country and year) life satisfaction-income gradient ($\hat{\text{β}}$) described by Equation 2. The unit of observation is a country × year. σ denotes the standard deviation of the component of the regression residual that is not due to sampling of the dependent variable, while ω represents the standard deviation of sampling error in the dependent variable. 95% confidence intervals in brackets. GDP = gross domestic product; FGLS = feasible generalized least square estimators.

*p < .05. **p < .01. ***p < .001.

In Table 5, we report tests of the income rank hypothesis using the other measures of subjective well-being. We observe that inequality appears to moderate the effect of income on optimism and enjoyment, while no effect was evident on measures of negative affect, such as anger, stress, and worry.

**Table 5.** Relation Between Income Inequality and Beta Coefficients for Optimism, Enjoyment, Anger, Stress, and Worry.

Dependent Variables	All countries	GDP/cap quartile 1	GDP/cap quartile 2	GDP/cap quartile 3	GDP/cap quartile 4
	(1)	(2)	(3)	(3)	(4)
	FGLS	FGLS	FGLS	FGLS	FGLS
*DV:* ${\text{β}}_{\text{ln}(income)}$ *predicting life satisfaction*					
Gini index	–0.980***	–1.432**	–1.236*	–1.378*	0.439
	[–1.489, –0.471]	[–2.306, –0.558]	[–2.408, –0.0653]	[–2.414, –0.342]	[–1.497, 2.375]
*DV:* ${\text{β}}_{\text{ln}(income)}$ *predicting optimism*					
Gini index	–1.344***	–1.555**	–1.801*	–1.146	–0.127
	[–1.920, –0.768]	[–2.618, –0.492]	[–3.143, –0.459]	[–2.505, 0.214]	[–2.426, 2.172]
*DV:* ${\text{β}}_{\text{ln}(income)}$ *predicting enjoyment*					
Gini index	–0.188***	–0.206*	–0.238*	–0.300***	0.195
	[–0.274, –0.102]	[–0.386, –0.0270]	[–0.426, –0.0495]	[–0.449, –0.151]	[–0.0389, 0.429]
*DV:* ${\text{β}}_{\text{ln}(income)}$ *predicting anger*					
Gini index	0.0509	0.0425	0.136	–0.00444	–0.0939
	[–0.00317, 0.105]	[–0.0664, 0.151]	[–0.00704, 0.279]	[–0.107, 0.0983]	[–0.236, 0.0486]
*DV:* ${\text{β}}_{\text{ln}(income)}$ *predicting stress*					
Gini index	0.0519	0.0499	0.149	0.110	–0.187
	[–0.0164, 0.120]	[–0.0841, 0.184]	[–0.00733, 0.306]	[–0.0136, 0.233]	[–0.377, 0.00363]
*DV:* ${\text{β}}_{\text{ln}(income)}$ *predicting worry*					
Gini index	0.0622	0.130	0.0762	0.0708	–0.0312
	[–0.0194, 0.144]	[–0.0786, 0.338]	[–0.0745, 0.227]	[–0.0620, 0.204]	[–0.322, 0.259]

*Note*. Columns show the marginal effects of Gini on other β coefficients (predicting optimism, enjoyment, anger, stress, and worry). All FGLS estimators control for a degree-three polynomial of GDP/cap and FEs for the surveys’ years. The unit of observation is a country × year. Estimators’ standard errors are clustered by country. 95% confidence intervals in brackets. GDP = Gross domestic product; FGLS = feasible generalized least square estimators.

*p < .05. **p < .01. ***p < .001.

As a final test of robustness, we repeated the main analysis with additional country-level covariates that might be confounded with inequality. Specifically, we added as covariates (a) the Gallup dataset’s *Community Basics Index*, which reflects the citizens’ evaluation of housing and infrastructure (public transportation, educational system, and healthcare system); (b) its *National Institutions Index,* which reflects confidence in key institutions (the military, the judicial system and the national government); and (c) its *Corruption Index,* which measures perceptions about the level of corruption in business and government. Table A6 in the Supplemental Material describes the survey questions and methodology used in their calculation. Index scores (in the range 0-100) are calculated at the individual record level. We computed final country-level index scores using the median of all individual records for each country and wave (country-level weights were applied to this calculation). Table 6 presents the results. We include these measures in separate specifications because they are highly correlated. The Gini coefficients in Columns 2, 3, and 4 were very similar to those obtained in our main analysis (Column 1), providing some reassurance that our key effects of Gini did not reflect a failure to include these covariates. Similar results were found using the income coefficient of variation instead of Gini measures (Table 7).

**Table 6.** Relation Between Income Inequality (Gini) and the Within-Country Life Satisfaction-Income Gradient, Robustness Test With additional covariates.

Variables	All countries			
	(1)	(2)	(3)	(4)
	FGLS	FGLS	FGLS	FGLS
Gini Index (0-1 scale)	–0.980***	–0.971***	–0.999***	–1.005***
	[–1.489, –0.471]	[–1.472, –0.471]	[–1.521, –0.478]	[–1.515, –0.495]
GDP/cap (in US$10,000 − 2011 PPP)				
GDP/cap	0.168*	0.164*	0.152*	0.124
	[0.0366, 0.299]	[0.0317, 0.297]	[0.0182, 0.285]	[–0.0199, 0.267]
GDP/cap^2^	–0.0659**	–0.0641**	–0.0603**	–0.0512*
	[–0.110, –0.0223]	[–0.107, –0.0208]	[–0.104, –0.0169]	[–0.0966, –0.00578]
GDP/cap^3^	0.00532**	0.00519**	0.00489**	0.00420*
	[0.00161, 0.00904]	[0.00151, 0.00887]	[0.00126, 0.00852]	[0.000495, 0.00791]
Community basics index (0-100 scale)		–0.000679		
		[–0.00420, 0.00284]		
Corruption index (0-100 scale)			0.000158	
			[–0.00122, 0.00153]	
National institutions index (0-100 scale)				–0.000766
				[–0.00251, 0.000981]
Constant	0.786***	0.774***	0.791***	0.851***
	[0.523, 1.050]	[0.433, 1.115]	[0.518, 1.065]	[0.571, 1.131]
Year *FE*	Yes	Yes	Yes	Yes
Observations	298	294	290	280
*R* ^2^	.133	.134	.131	.142
σ	.200	.202	.201	.192
ω	.106	.106	.107	.107

*Note*. Columns show Feasible Generalized Least Square Estimators (FGLS) with standard errors clustered by country. The dependent variable is the (within country and year) life satisfaction-income gradient ($\hat{\text{β}}$) described by Equation 2. The unit of observation is a country × year. σ denotes the standard deviation of the component of the regression residual that is not due to sampling of the dependent variable, while ω represents the standard deviation of sampling error in the dependent variable. 95% confidence intervals using clustered standard errors by country in brackets. FGLS = feasible generalized least square estimators; GDP = gross domestic product.

*p < .05. **p < .01. ***p < .001.

**Table 7.** Relation Between Income Inequality (Coefficient of Variation for Income) and the Within-Country Life Satisfaction-Income Gradient, Robustness Tests.

Variables	All countries			
	(1)	(2)	(3)	(4)
	FGLS	FGLS	FGLS	FGLS
Coefficient of variation	−0.138***	−0.138***	−0.158***	−0.154***
	[−0.204, −0.0726]	[−0.203, −0.0735]	[−0.219, −0.0978]	[−0.213, −0.0950]
GDP/cap (in US$10,000 − 2011 PPP)				
GDP/cap	0.0778	0.0729	0.0428	0.0182
	[−0.0513, 0.207]	[−0.0571, 0.203]	[−0.0925, 0.178]	[−0.121, 0.157]
GDP/cap^2^	−0.0244	−0.0218	−0.0124	−0.00438
	[−0.0643, 0.0155]	[−0.0615, 0.0180]	[−0.0552, 0.0303]	[−0.0463, 0.0375]
GDP/cap^3^	0.00157	0.00139	0.000637	0.0000312
	[−0.00163, 0.00477]	[−0.00180, 0.00457]	[−0.00272, 0.00399]	[−0.00321, 0.00327]
Community basics index (0-100 scale)		−0.00123		
		[−0.00461, 0.00215]		
Corruption index (0-100 scale)			0.000277	
			[−0.00117, 0.00172]	
National institutions index (0-100 scale)				−0.000947
				[−0.00261, 0.000717]
Constant	0.516***	0.609***	0.581***	0.647***
	[0.385, 0.647]	[0.315, 0.903]	[0.362, 0.800]	[0.387, 0.906]
Year *FE*	Yes	Yes	Yes	Yes
Observations	284	280	277	267
*R* ^2^	.121	.124	.131	.142
σ	.199	.201	.199	.189
ω	.108	.108	.109	.109

*Note*. Columns show FGLS with standard errors clustered by country. The dependent variable is the (within country and year) life satisfaction-income gradient ($\hat{\text{β}}$) described by Equation 2. The unit of observation is a country × year. σ denotes the standard deviation of the component of the regression residual that is not due to sampling of the dependent variable, while ω represents the standard deviation of sampling error in the dependent variable. 95% confidence intervals using clustered standard errors by country in brackets. FGLS = feasible generalized least square estimators.

*p < .05. **p < .01. ***p < .001.

### Discussion

The results of Study 2 provide further evidence that, as predicted by the income rank hypothesis, the relationship between life satisfaction and income is moderated by inequality across different countries. More specifically, and as in Study 1, in more equal countries a given increase in income leads a greater increase in life satisfaction. Comparing as in Study 1 countries at the 5th and 95th percentiles of income inequality, the effect of a 10% increase in income on life satisfaction was 1.65 times larger for low-inequality countries.

The result was robust to the inclusion of both country-level and individual-level controls and was also robust to the use of a different measure of income inequality. Similar effects were found with some other measures of subjective well-being. We also found main effects of both income and Gini on life satisfaction, but as these effects have both been examined extensively in previous literature we do not consider them further.

## General Discussion

The primary aim of the research reported here was to test a novel prediction of the income rank hypothesis. Specifically, it was predicted that the increase in self-reported life satisfaction that results from a given increase in income would be larger in countries in which incomes were more equally distributed. The prediction was confirmed in two studies each of which used a different dataset. Moreover, the results were robust to inclusion of individual-level and country-specific characteristics and alternative measures of income inequality.

In this general discussion, we first discuss the theoretical implications of the results in the context of the income rank hypothesis and in relation to other sources of support for that hypothesis. We also show how the findings cause difficulty for conventional economic approaches. After a brief consideration of limitations and generality, we then discuss how the present findings and the income rank hypothesis relate to the wider literature on the psychology of income inequality.

### Theoretical Implications

First, while noting the importance of many other influences on life satisfaction (Diener & Seligman, 2004; Inglehart et al., 2008), we interpret the results in terms of the hypothesis that self-reported life satisfaction derives at least in part from the relative social rank that income confers—that is, the income rank hypothesis. The results therefore sit well with a range of other related findings that have been taken to implicate the importance of income rank. We have already noted that rank of income, rather than income, predicts a number of facets of subjective well-being. These results are in turn consistent with the well-established ideas that people engage in social comparison and are concerned with social status. The income rank hypothesis also fits well with the observation of absent or at least small or inconsistent effects of income inequality on mean society-level well-being.

In contrast, our results are inconsistent with the assumptions of conventional economic approaches in at least two related ways. First, we have shown that the assumption of a fixed relationship between income and life satisfaction (e.g., Stevenson & Wolfers, 2013) is wrong. We found instead that society-level income inequality strongly moderates the relationship. To the extent that well-being proxies utility (Oswald & Wu, 2010), the results suggest that the slopes of utility curves are not stable but depend on underlying income distributions. Second, the income rank hypothesis may illuminate other consequences of income inequality that appear to run counter to conventional economic models. The income rank hypothesis account predicts concave income-utility functions whenever incomes are positively skewed (Brown et al., 2008; Stewart et al., 2006) because, as one moves up the income scale, ever higher increments of income are needed to buy a fixed increment in ranked position within the skewed distribution. However, the income rank account of the diminishing marginality utility of income makes a different prediction from the standard account for the effects of inequality on aggregate well-being within a country. According to a conventional model in which income has a positive but diminishing marginal impact on utility, country-level income inequality should have a negative influence on average well-being within a country (Lerner, 1944). The income rank hypothesis, in contrast to the conventional approach, predicts no effect of income inequality on mean satisfaction—because the mean relative income rank will always be 0.5, no matter how the income is distributed.

In summary, the income rank hypothesis predicts (a) a concave relationship between income and life satisfaction in individual countries, along with (b) absent or at least small or inconsistent effects of income inequality on mean society-level well-being and (c) steeper income/well-being gradients in more equal countries. These predictions are, we suggest, largely consistent with the observed data, despite the undoubted importance of many other variables not examined here.

### Limitations and Generality

The relationships we have reported here are correlational. It is therefore possible that causality runs from income/well-being gradients to societal income inequality. Perhaps some societies are composed of individuals who gain greater well-being increases from income increments, and such individuals vote for redistributive tax and welfare policies. Although our data cannot exclude such a possibility, it seems unlikely. A longitudinal analysis—showing that changes in inequality lead to subsequent changes in the gradients linking income to well-being—is desirable but difficult in practice, partly because of collinearity issues and partly because of inevitable confounding factors, such as political climate and other economic variables, which render it difficult to isolate time-varying effects of inequality per se.

Our ability to control for potential confounding variables is inevitably limited by the datasets available to us. We are, therefore, unable to alleviate concerns of omitted variable bias completely; such reassurance will require experimental testing. We were, however, able to include a number of individual-level and country-level controls, some in Study 1 and others in Study 2, and our key result survived the inclusion of all such control variables.

We also note the variety of different measures that have been used in our analysis. In Study 1, the dependent variable of interest was a standard measure of life satisfaction. This is conventionally interpreted as a measure of subjective well-being, as it asks the responder about their mental state. We also found evidence for the income rank hypothesis when the dependent variable was either optimism or enjoyment (Study 2). However, we also found the result with measures of self-reported social class (Study 1) and self-reports of position on a ladder where the top represents “the best possible life for you” and the bottom represents “the worst possible life.” Although the “ladder” item is often interpreted as measuring life satisfaction, the ladder items ask individuals for an evaluation of their objective life circumstances rather than asking about their mental states directly. The income rank hypothesis, therefore, receives support from a range of independent variables which differ in how directly they probe participants’ mental states.

A further potential limitation arises from our assumption that rank alone influences life satisfaction. The income rank assumption assumes that (a) incomes higher and lower than the income of an individual carry equal weight in determining that individual’s life satisfaction, and (b) that all incomes are equally weighted irrespective of how far away they are from the relevant individual’s own income. However, income rank can be seen as a special case of a more general metric (Brown et al., 2008; Hounkpatin et al., 2020), and future research will be needed to explore whether the improved fit of a more general model (with additional parameters) is sufficient to justify such a model’s additional complexity.

### Relation to Wider Literature

Although the present results are as predicted by the income rank hypothesis, they may at first blush appear more difficult to reconcile with wider claims in the psychological literature on income inequality. Specifically, our results show that an individual living in an equal society requires a smaller increase in income to achieve a one-point increase in life satisfaction than would be required if that same individual lived in a less equal society. One might therefore assume that people would devote more of their attention to increasing their incomes if they lived in more equal societies, because the resulting increase in their life satisfaction would be correspondingly greater. Put another way, it could plausibly be hypothesized that when increments in social rank are more expensive to obtain, as they appear to be in more unequal societies, rational agents would devote more of their resources to obtaining alternative goods (such as leisure or the development and maintenance of protective social networks) if utility comes from rank itself rather than the associated material position (Hopkins, 2008). However, a large body of research suggests that in fact people devote *more* attention to achieving success in material aspects of life when inequality is high, the tendency of married partners to have similar incomes has increased greatly as inequality has risen (Milanovic, 2019), and people’s subjective well-being is more strongly influenced by the income of their neighbors when inequality is high (Cheung & Lucas, 2016). Such results seem to suggest (consistent with intuition) *less* concern with income maximization in more equal societies. Other research suggests that income inequality is associated with increased materialism, social comparison, and status anxiety as well as reduced trust (for reviews, see, for example, Buttrick et al., 2017; Walasek & Brown, 2019; Wilkinson & Pickett, 2018). For example, income inequality is associated with increased Internet searching for, and tweeting about, positional/status goods such as designer brands (Walasek et al., 2018; Walasek & Brown, 2015, 2016), although it is unclear whether the increased concern with status and comparison applies in all domains of life or only with regard to material aspects (Walasek & Brown, 2019).

How can these two sets of findings be reconciled? On the one hand, the income rank hypothesis suggests that effort devoted to increasing one’s income would bring greater returns (at least in terms of subjective life satisfaction) in more equal societies. On the other hand, people seem to concern themselves more with income and wealth-related activities in more unequal societies. Although provision of a complete model lies outside the scope of the present paper, we note here a number of ways in which this apparent tension may be resolved while making the assumption that, while social comparison processes are likely to be important in any account, the nature of such comparisons and their relation to self-reported life satisfaction may vary as a function of inequality.

One possibility is simply that people are influenced by the fact that increments in income rank are associated with greater absolute material gains (and hence are more worth pursuing) when inequality is high, although such an account would go against the well-evidenced idea that people care more about relative than absolute income. An alternative possibility is that fixed increments of income are more difficult (e.g., require more effort) to obtain in more equal societies, and that this increased difficulty either outweighs the potential increases in life satisfaction that could be obtained, or would involve a concomitant reduction in other aspects of subjective well-being.

A third possibility is that people will care more about income and wealth in a more unequal society because income is a more reliable signal of social status in such societies. Specifically, one hypothesis is that inequality influences the relative weights given to social comparisons that concern income and material characteristics as opposed to social comparisons that concern less materialistic characteristics (see Walasek et al., 2018; Walasek & Brown, 2015 for discussion). If that is the case, it would not be surprising if individuals in an unequal society were prepared to work longer hours, sacrificing other goods such as leisure activities and the development and maintenance of possibly protective social networks and health behaviors, to maximize their income. Consistent with such a perspective, there is ample evidence that working hours are longer in more unequal societies (e.g., Bowles & Park, 2005), and that there is less trust (e.g., Oishi et al., 2011), lower agreeableness (de Vries et al., 2011), and more cheating (e.g., Neville, 2012) in societies where income is more unequally distributed. Moreover, characteristics such as facial masculinity, which may be positively associated with aggression and dominance of the type that may predict success in competitive environments but negatively associated with parental investment, are preferred by females more strongly when inequality is high (Brooks et al., 2011).

Finally, it is possible that people have uncertainty about their preferences (e.g., for materialist behavior and social status relative to other aspects of life), and that their beliefs about their preferences are, therefore, influenced by the social norm (here, simply the observable behavior of others). More specifically, people may as adolescents or young adults be forming their beliefs about their own preferences and life goals. These beliefs will based partly on people’s private signals about their own preferences, but (to the extent that people believe they are similar to other people) should also be influenced by observation of other people’s preferences as reflected in their life choices. If one inhabits a society in which levels of materialism and concern for income-related social comparison are high, it is rational to adjust one’s beliefs about one’s own preferences in that direction.

In sum, there are several ways in which the income rank hypothesis may be reconciled with evidence for increased concern with status and social comparison in more unequal societies. Further research will be needed to adjudicate between these accounts.

## Supplemental Material

Supplemental Material, Quispe-Torreblanca_Online_appendix - Inequality and Social Rank: Income Increases Buy More Life Satisfaction in More Equal Countries Click here for additional data file. Supplemental Material, Quispe-Torreblanca_Online_appendix for Inequality and Social Rank: Income Increases Buy More Life Satisfaction in More Equal Countries by Edika G. Quispe-Torreblanca, Gordon D. A. Brown, Christopher J. Boyce, Alex M. Wood and Jan-Emmanuel De Neve in Personality and Social Psychology Bulletin

## Notes

1. Although “utility” is normally interpreted as a derived theoretical quantity, whereas subjective well-being is a mental state, we follow a large existing literature in assuming a relationship between the two.

2. Although we do not have control of the sample size, power calculation showed that the sample size required to detect an increase in *R* ^2^ by 10% after adding the Gini coefficient in the second step of our two-step estimation procedure, with 80% power using a 5% level test, is approximately 64 observations (here, countries).

3. We thank them for this.

4. This initial approximation was omitted from Study 1 because WVS registers income in different currencies, while the Gallup survey registers annual income expressed in international dollars. While we could add country fixed effects to account for the differences in currencies across countries, because Gini is a country-level variable it would be perfectly collinear to the country fixed effects, making unfeasible the identification of the main effect of inequality on life satisfaction.
