# Supplementary material for: Inequality and Social Rank: Income Increases Buy More Life Satisfaction in More Equal Countries
Source: Pers Soc Psychol Bull. 2020 May 29;47(4):519–39. doi: 10.1177/0146167220923853 (PMC7961663; doi:10.1177/0146167220923853)
Supplement: Supplemental Material, Quispe-Torreblanca_Online_appendix - Inequality and Social Rank: Income Increases Buy More Life Satisfaction in More Equal Countries [file Quispe-Torreblanca_Online_appendix.pdf]

Methods: Survey Questions

Life Satisfaction World Values Survey Questions

Variable	Range	Question
Life Satisfaction	1 to 10	All things considered, how satisfied are you with your life as a whole these days?, where 1 means you are "completely dissatisfied" and 10 means you are "completely satisfied" (A170)
Subjective Social Class	1 to 5	People sometimes describe themselves as belonging to the working class, the middle class, or the upper or lower class. Would you describe yourself as belonging to the: 1 Upper class, 2 Upper middle class, 3 Lower middle class, 4 Working class, 5 Lower class (X045)

Life Satisfaction Gallup Survey Questions

Variable	Range	Question
Current Life Satisfaction	0 to 10	Please imagine a ladder, with steps numbered from 0 at the bottom to 10 at the top. The top of the ladder represents the best possible life for you and the bottom of the ladder represents the worst possible life for you. On which step of the ladder would you say you personally feel you stand at this time? (WP16)
Expected Life Satisfaction (Optimism)	0 to 10	Please imagine a ladder, with steps numbered from 0 at the bottom to 10 at the top. The top of the ladder represents the best possible life for you and the bottom of the ladder represents the worst possible life for you. Just your best guess, on which step do you think you will stand in the future, say about five years from now? (WP18)
Enjoyment	0 to 1	Did you experience the following feelings during a lot of the day yesterday? How about enjoyment? (WP67)
Anger	0 to 1	Did you experience the following feelings during a lot of the day yesterday? How about anger? (WP74)
Stress	0 to 1	Did you experience the following feelings during a lot of the day yesterday? How about stress? (WP71)
Worry	0 to 1	Did you experience the following feelings during a lot of the day yesterday? How about worry? (WP69)

Institutions and Infrastructure Indexes, Gallup Survey Questions

Variable	Range	Question
Community Basics Index	0 to 100	In the city or area where you live, are you satisfied or dissatisfied with the public transportation systems? (WP91)<br>In the city or area where you live, are you satisfied or dissatisfied with the roads and highways? (WP92)<br>In your city or area where you live, are you satisfied or dissatisfied with the quality of air? (WP94)<br>In your city or area where you live, are you satisfied or dissatisfied with the quality of water? (WP95)<br>In your city or area where you live, are you satisfied or dissatisfied with the availability of good affordable housing? (WP98)<br>In the city or area where you live, are you satisfied or dissatisfied with the educational system or the schools? (WP93)<br>In the city or area where you live, are you satisfied or dissatisfied with the availability of quality healthcare? (WP97)
National Institutions Index	0 to 100	Do you have confidence in each of the following, or not? How about the military? (WP137)<br>Do you have confidence in each of the following, or not? How about the judicial system and courts? (WP138)<br>Do you have confidence in each of the following, or not? How about the national government? (WP139)<br>Do you have confidence in each of the following, or not? How about the honesty of elections? (WP144)
Corruption Index	0 to 100	Is corruption widespread within businesses located in (country), or not? (WP145)<br>Is corruption widespread throughout the government in (country), or not? (WP146)
